# Genetic Analysis of Signal Generation by the Rgt2 Glucose Sensor of *Saccharomyces cerevisiae*

**DOI:** 10.1534/g3.118.200338

**Published:** 2018-06-28

**Authors:** Peter Scharff-Poulsen, Hisao Moriya, Mark Johnston

**Affiliations:** * Department of Biology, University of Copenhagen, Copenhagen DK-2100, Denmark; † Department of Genetics, Washington University School of Medicine, 4566 Scott Avenue, St. Louis, MO 63110; ‡ Department of Biochemistry and Molecular Genetics, University of Colorado School of Medicine, Aurora, CO 80045

**Keywords:** glucose sensor, sugar transporter, glucose signaling

## Abstract

The yeast *S. cerevisiae* senses glucose through Snf3 and Rgt2, transmembrane proteins that generate an intracellular signal in response to glucose that leads to inhibition of the Rgt1 transcriptional repressor and consequently to derepression of *HXT* genes encoding glucose transporters. Snf3 and Rgt2 are thought to be glucose receptors because they are similar to glucose transporters. In contrast to glucose transporters, they have unusually long C-terminal tails that bind to Mth1 and Std1, paralogous proteins that regulate function of the Rgt1 transcription factor. We show that the C-terminal tail of Rgt2 is not responsible for its inability to transport glucose. To gain insight into how the glucose sensors generate an intracellular signal, we identified *RGT2* mutations that cause constitutive signal generation. Most of the mutations alter evolutionarily-conserved amino acids in the transmembrane spanning regions of Rgt2 that are predicted to be involved in maintaining an outward-facing conformation or to be in the substrate binding site. Our analysis of these mutations suggests they cause Rgt2 to adopt inward-facing or occluded conformations that generate the glucose signal. These results support the idea that Rgt2 and Snf3 are glucose receptors that signal in response to binding of extracellular glucose and inform the basis of their signaling.

Yeast cells sense glucose through two membrane proteins, Rgt2 and Snf3, which generate an intracellular signal that ultimately inhibits function of the Rgt1 transcriptional repressor, leading to de-repression of expression of *HXT* genes encoding hexose transporters (reviewed in (Ozcan and Johnston 1999). The intracellular signal generated by the Rgt2 and Snf3 glucose sensors results in phosphorylation of Mth1 and Std1, two paralogous regulators of Rgt1 that are necessary for its repressor function (Moriya and Johnston 2004). Phosphorylation of Mth1 and Std1 renders them substrates of the SCF^Grr1^ ubiquitin-protein ligase, which catalyzes their ubiquitination, thereby targeting them for degradation by the proteasome (Flick *et al.* 2003; Kim *et al.* 2003; Lakshmanan *et al.* 2003; Mosley *et al.* 2003). The loss of Mth1 and Std1 robs Rgt1 of proteins required for its repressor function, resulting in derepression of *HXT* gene expression (Flick *et al.* 2003; Kim *et al.* 2003; Lakshmanan *et al.* 2003; Mosley *et al.* 2003; Polish *et al.* 2005).

The glucose sensors are structurally similar to glucose transporters, with 12 predicted transmembrane spanning segments. They differ from all known glucose transporters in their unusually long C-terminal tails, which are predicted to extend into the cytoplasm (Ozcan *et al.* 1996). These tails bind to Mth1 and Std1 and contribute to the glucose signaling activity of the sensors (Ozcan *et al.* 1998; Schmidt *et al.* 1999; Lafuente *et al.* 2000). The transmembrane domain by itself can generate the glucose signal if overexpressed, suggesting that the C-terminal tails of the sensors serve to enhance signaling (Moriya and Johnston 2004). Our current view is that binding of glucose to the glucose sensors changes their conformation, leading to the phosphorylation of Mth1 and Std1, which are bound to the C-terminal tails of the sensors. High intracellular glucose levels inhibit signaling through Snf3 (Karhumaa *et al.* 2010), suggesting that Snf3 and Rgt2 sense both intracellular and extracellular glucose.

Rgt2 and Snf3 are related to sugar porters that are members of the major facilitator superfamily (MFS), which mediate transport of sugars by facilitated diffusion or proton symport. The structures of some sugar porters—XylE, GlcP, GLUT1, GLUT3 and GLUT5—have been determined (Sun *et al.* 2012; Iancu *et al.* 2013; Quistgaard *et al.* 2013; Deng *et al.* 2014; Wisedchaisri *et al.* 2014; Deng *et al.* 2015; Nomura *et al.* 2015; Kapoor *et al.* 2016). These transporters have 12 transmembrane (TM) helices, with N-terminal and C-terminal halves, each consisting of six transmembrane helices, connected by a cytosolic loop with four intracellular helices (ICHs), and a fifth ICH following TM12. Transport of sugars is achieved by a rocker-switch alternating-access mechanism in which the two bundles of TM helices move around a central substrate-binding site, making the substrate binding pocket accessible to either the outside or the inside of the cell (reviewed in Yan 2015; Drew and Boudker 2016; Yan 2017). The global rearrangement of the two six-helix bundles is accompanied by local rearrangements of gating helices TM1 and TM7, which make extracellular cavity-closing contacts, and gating helices TM4 and TM10, which make intracellular cavity-closing contacts.

The current model of the sugar porter family has the transporter maintained in an outward-facing conformation in the absence of substrate by a network of inter-bundle salt-bridges located at the cytoplasmic side. The intracellular helices (ICH1-5) contribute to stabilizing the outward-facing conformation through polar interactions and salt bridges between the ICHs and the TMs (Sun *et al.* 2012; Nomura *et al.* 2015). Substrate binding disrupts the inter-bundle salt-bridge network and the TM-ICH interactions, resulting in an inward-open conformation and release of substrate inside the cell.

It is not clear how the glucose sensors become activated upon glucose binding. In an attempt to illuminate this process, we carried out a genetic analysis of the Rgt2 glucose sensor. We analyzed the role of the C-terminal tail of Rgt2 in glucose signaling and glucose transport. To address the issue of the role of the transmembrane domains in generating a glucose signal we identified mutations of *RGT2* that cause constitutive signaling. We use the structures of sugar porters to model how the mutations affect the Rgt2 structure in order to inform its signaling conformation(s).

## Materials and Methods

### Strains and media

Yeast strains used in this study are: **YM6212**: *MATα ura3-52 his3-200 ade2-101 lys2-801 trp1-903 leu2-3,112 tyr1-501 snf3*::*HIS3rgt2*::*HIS3*; **YM6453***: MATa ura3-52 his3-200 ade2-101 lys2-801 leu2trp1-903 tyr1-501 TRP1*::*HXT3promoter-HIS3*; **EBY.VW1000***: MATα leu2-3,112 ura3-52 trp1-289 his3-Δ1 MAL2-8 hxt5,1,4Δ*::*loxP hxt3,6,7Δ*::*loxP hxt2Δ*::*loxP hxt8Δ*::*loxP hxt9Δ*::*loxP hxt10Δ*::*loxP hxt11Δ*::*loxP hxt12Δ*::*loxP hxt13Δ*::*loxP hxt14Δ*::*loxP hxt15Δ*::*loxP hxt16Δ*::*loxP hxt17Δ*::*loxP gal2Δ (*Wieczorke *et al.* 1999). Cells were grown either on YP (2% bacto-peptone, 1% yeast extract (Difco) or YM (0.67% yeast nitrogen base (Difco) plus 0.5% ammonium sulfate lacking the appropriate amino acids) medium, supplemented with either 2% glucose (YPD) or 2% raffinose or 2% maltose, or 5% glycerol + 2% ethanol (YMGE). The *E. coli* strain DH5α was used as host for plasmids.

### Plasmids

Plasmids used in this study are listed in Table 1. Unless otherwise stated, DNA fragments used in plasmid constructions were amplified by the PCR with the Expand High Fidelity PCR System (Roche Molecular Biochemicals), and cloned into plasmids by gap repair (Oldenburg *et al.* 1997). Mutagenesis of full length *RGT2* was performed using pBM3868 as template.

**Table 1:** Plasmids

Plasmid	Relevant components	Source or reference
pBM2636	*URA3*-p*HXT1*::*lacZ* in Yep357R	(Ozcan and Johnston 1995)
pBM2974	*ADH1* promoter in pRS426	(Ozcan *et al.* 1998)
pBM4436	*ADH1* promoter-*HXT1* in pRS426	This study
pBM4584	*ADH1* promoter-*HXT1*-D82V in pRS426	This study
pBM4585	*ADH1* promoter-*HXT1*-I192Q in pRS426	This study
pBM4586	*ADH1* promoter-*HXT1*-D382N in pRS426	This study
pBM4587	*ADH1* promoter-*HXT1*-L521S in pRS426	This study
pBM4528	*ADH1* promoter*-RGT2* in pRS426	(Moriya and Johnston 2004)
pBM4590	*ADH1* promoter*-RGT2∆*tail-HXT1 tail ver. “a” in pRS426	This study
pBM4531	*ADH1* promoter*-RGT2∆*tail-HXT1 tail ver. “b” in pRS426	(Moriya and Johnston 2004)
pBM4591	*ADH1* promoter*-HXT1-RGT2*tail ver. “a” in pRS426	This study
pBM4529	*ADH1* promoter*-HXT1-RGT2*tail ver. “b” in pRS426	(Moriya and Johnston 2004)

#### Construction of plasmids used in Hxt1-Rgt2 chimera analysis:

**Figure 1:**
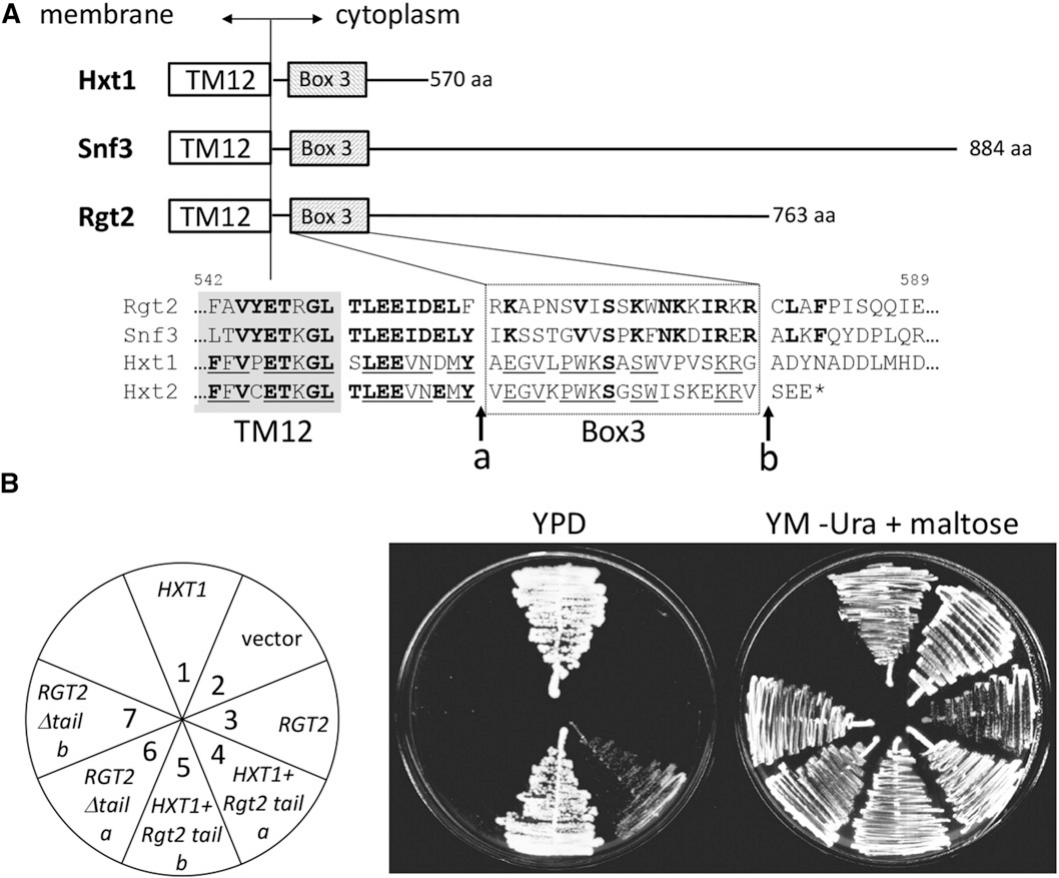
The glucose sensor tails do not influence glucose transport. A) A CLUSTALW alignment of the sequences immediately adjacent to the last predicted transmembrane domain of the glucose sensors and two glucose transporters (TM12, enclosed in the box) is shown. Conserved amino acids are shown in bold; amino acids conserved only among glucose transporters are underlined. The Rgt2 sensor tail was fused to Hxt1 or deleted from Rgt2 at the “a” and “b” junctions indicated by the arrows. B) Glucose transport activity of indicated chimeras. Strain EBY.VW1000 carrying plasmids pBM2974 (vector), pBM4436 (*HXT1*), pBM4528 (*RGT2*), pBM4591 (*HXT1-Rgt2 tail “a*”), pBM4529 (*HXT1-Rgt2 tail “b*”), pBM4590 (*RGT2Δtail “a*”), pBM4531 (*RGT2Δtail “b*”) were streaked on YPD (2% glucose) plates and YM-uracil plates with 2% maltose.

pBM4529: Hxt1-Rgt2tail version “b”, which consists of the transmembrane domain of Hxt1 (amino acids 1-547) and the C-terminal tail of Rgt2 (amino acids 579-end), has been described (Moriya and Johnston 2004); pBM4531: Rgt2Δtail version “b” (see Figure 1A), consisting of Rgt2 (amino acids 1-578) and the Hxt1 C-terminus (amino acids 548-end) was previously described (Moriya and Johnston 2004); pBM4591, the transmembrane domain of Hxt1 (amino acids 1–528) and the Rgt2 C-terminal tail (amino acids 560–end) were fused and cloned into pBM2974; pBM4590: the Rgt2 transmembrane domain (amino acids 1–559) and the C-terminus of Hxt1 (amino acids 529–end) were fused and cloned into pBM2974.

### Measurement of glucose induction of *HXT1* or *HXT3* gene expression

For the experiment reported in Table 3, cells of the indicated yeast strains containing the *HXT1-lacZ* plasmid (pBM2636) were grown overnight in media selective for the plasmids with 0.5% galactose + 5% glycerol. Cells were diluted into YM containing 4% glucose, grown at 30° to log phase (OD_600_ ∼1), and their β-galactosidase activity was measured. For the experiment reported in Table 4, cells carrying the *HXT1-lacZ* plasmid (pBM2636) and the indicated *RGT2* mutation were grown to log phase on 5% glycerol + 0.5% galactose and assayed for their β-galactosidase activity. β-galactosidase activity assays were performed using the Yeast β-galactosidase assay kit (Pierce, Rockford, IL) according to the manufacturer’s instructions. Results are calculated in Miller units ((1000x OD_420_)/(T x V x OD_600_) T = incubation time in min., V = volume of cells in ml).

### Isolation of constitutively signaling *RGT2* mutants

*RGT2* was mutagenized by its amplification in a “dirty” PCR using pBM3868 as template, as described by (Xu *et al.* 1999), then cloned into pRS316 (Sikorski and Hieter 1989) by gap-repair through transformation of YM6453, which contains an *HXT3-HIS3* reporter integrated at *TRP1*. Transformants were selected on YM-Ura plates, then replica plated onto YM-Ura-His plates containing 5% glycerol and 2% ethanol (YMGE-Ura-His). His^+^ colonies growing on these plate were mated with YM4128 harboring pBM2636 (*HXT1-lacZ*), then selected again on YMGE-Ura-His plates. The resulting strains were assayed for their β-galactosidase activity to confirm their constitutive expression of *HXT1*. Plasmids from the strains exhibiting strong β-galactosidase activity in YMGE medium (about 10 fold higher than wild type) were recovered in *E. coli* and the nucleotide sequence of *RGT2* was determined (the). The *RGT2* mutation(s) responsible for the constitutive phenotype were identified as follows:

1. When a mutant contains only one *RGT2* mutation, the mutation was assumed to cause the constitutive activity (R231K in *RGT2-1*, D415N in #47, W474R in #60, V475M in #95, Q222I in #83 and F113L in #87 and #93).
2. For *RGT2* mutants that contained multiple mutations, one of which is one of the mutations identified in (1), and when it does not contain a mutation isolated more than once in this screen, we assume the mutation causing constitutive activity is the same as the one identified in (1) (R231G in #98 and #50, F113L in #58 and #78).
3. For *RGT2* mutants that contained multiple mutations, none of which were found in (1), we dissected the mutations by amplifying a portion of *RGT2* containing each single mutation and replacing these sequences of wild type *RGT2* by gap-repair. Each plasmid with a single amino acid substitution was transformed into YM4128 harboring pBM2636 (*HXT1-lacZ*) and its effect on β-galactosidase activity determined. Mutations identified in this way are D116V in #82, G232S in #59, S236P in #81, L552S in #52. In the case of #49, both the S253P and S443P mutations seem to contribute to constitutive signaling, because each causes a low level of constitutive β-galactosidase activity. We did not attempt to dissect the mutation in mutants #108 and #92 because they are closely linked.
4. The remaining mutants resulted in amino acid changes in the same residues identified in (3) (D116V in #68, D116G in #57 and #111, D116N in #100, G232S in #59, S236P in #81, L552S in #111 and #85). Because the other mutations in those *RGT2* mutants were isolated only once, it is likely that those mutations are not relevant to the constitutive activity (F465Y was tested by site directed mutagenesis and found not to cause constitutive signaling (data not shown)).

### Assessment of glucose transport function

Plasmids expressing Hxt1 with amino acids substitutions were constructed by the gap-repair method using two PCR products amplified from pBM4527 (*ADH1promoter-HXT1*) as a template. These *HXT1* plasmids were introduced into EBY.VW1000 (*hxt* null mutant) (Wieczorke *et al.* 1999) and the transformants were selected on YM-Ura containing 2% maltose, then streaked on YPD (to assess their ability to grow on glucose) or on YM-Maltose-Ura (Figure 3).

### Bioinformatics

Amino acid sequence alignments were carried out using Clustal Omega (http://www.ebi.ac.uk/Tools/msa/clustalo/) and ESPript 3 at http://espript.ibcp.fr/ESPript/ESPript/ (Robert and Gouet 2014). Remote homology detection and building of 3D protein structure models was carried out on the Phyre2 web portal at http://www.sbg.bio.ic.ac.uk/phyre2/html/page.cgi?id=index (Kelley *et al.* 2015). Protein topology models of Rgt2 were made on Protter at http://wlab.ethz.ch/protter/start/ (Omasits *et al.* 2014). 3D protein structure figures were prepared using PyMol (Schrödinger, LLC).

### Data and Reagent Availability

The authors affirm that all data necessary for confirming the conclusions of this article are represented fully within the article and its tables and figures. Strains are available upon request. Supplemental material available at Figshare: https://doi.org/10.25387/g3.6683558.

## Results

### The C-terminal tails of the glucose sensors are not involved in glucose transport

Rgt2 and Snf3 do not transport glucose (Ozcan *et al.* 1998), even though their transmembrane domains are similar to hexose transporters. It seemed possible that their C-terminal tails might be responsible for this functional difference between the glucose sensors and transporters. We therefore tested the role of the Rgt2 tail in glucose transport. We fused the Rgt2 tail to the transmembrane domains of Hxt1 (at junction “b” in Figure 1A) and expressed the chimeric protein in yeast strain EBY.VW1000 deleted for all its glucose transporters (Δ*hxt1-17* Δ*gal2*) but with functional maltose transporters (Wieczorke *et al.* 1999). The tail of Rgt2 does not inhibit the ability of Hxt1 to transport glucose (sector 5 of Figure 1B), suggesting that the sensor tail is not responsible for inhibiting glucose transport activity.

The 19 amino acids adjacent to transmembrane domain 12 (TM12) of Hxt1 that we call Box 3 (Figure 1A) are conserved in the glucose sensors. The corresponding sequences of the glucose transporters are similar to each other, but different from those in the glucose sensors. Box 3 seems to be important for the glucose transport activity of Hxt1, because attaching the Rgt2 tail proximal to these sequences in Hxt1 (at junction “a” in Figure 1A), thereby deleting these sequences from Hxt1, reduces (but does not abolish) its ability to transport glucose (sector 4 in Figure 1B).

Conversely, removal of the tail from Rgt2 does not enable it to transport glucose (sectors 6 and 7 of Figure 1B). The Rgt2∆tail proteins appear to be functional, because they can generate a signal that induces *HXT1* expression (Table 3). (The tail-less Rgt2 proteins are expressed at high levels from the *ADH1* promoter, and we previously showed that the tail-less Rgt2 can generate a glucose signal if overexpressed (Moriya and Johnston 2004)).

### *RGT2* mutations causing constitutive signaling

**Figure 2:**
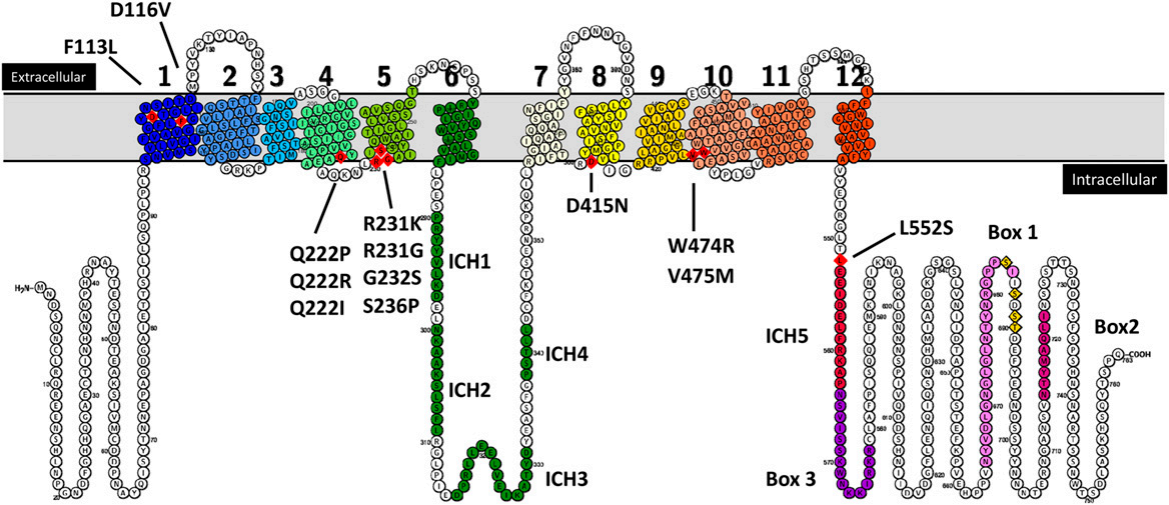
Location of *RGT2* mutations that cause constitutive glucose signaling. Rgt2 topology model in which the positions (red diamonds) of the various *RGT2* mutations are shown. The transmembrane helices (TMs) are numbered from 1 through 12 and shown in the color scheme used throughout the article. The intracellular helices (ICH1 to ICH4) between TM6 and TM7 are shown in green; ICH5 following TM12 is in red. Box 1, Box2 and Box 3 (violet dots) and potential phosphorylation sites (yellow diamonds) (Snowdon and Johnston 2016) are indicated.

Substitution of Arg-231 with Lys in Rgt2 and the orthologous amino acid substitution in Snf3 (Arg-229 to Lys) cause the glucose sensors to generate a constitutive glucose signal, presumably because they lock the sensors in their glucose bound conformation (Ozcan *et al.* 1996). Believing that additional mutations might illuminate the mechanism of glucose signal generation, we isolated other mutations that cause constitutive glucose signaling. *RGT2* on a low-copy plasmid was mutagenized in a “dirty PCR” (Xu *et al.* 1999), and the resulting library of plasmids was introduced into a strain carrying *HIS3* under the control of the *HXT3* promoter, and the transformants were screened for constitutive expression of *HXT3* (manifested by a His^+^ phenotype on plates without glucose; see Materials and Methods for details). Of approximately 200,000 transformants screened, 70 were His^+^ in the absence of glucose. Twenty-five of these mutants showed a significant increase in *HXT1-lacZ* expression over wild type after growth of cells on glycerol + ethanol, and their *RGT2* nucleotide sequences were determined. The 25 *RGT2* mutants proved to be independent, because they have different nucleotide substitutions (Table 2). Most mutants carried several mutations, but in each case one mutation was found to be responsible for constitutive glucose signaling (see Materials and Methods for how this was determined). Ten different amino acid positions are affected by these mutations, 9 of which are predicted to lie in transmembrane helixes of Rgt2; one lies adjacent to intracellular helix 5 (ICH5) (Table 4 and Figure 2).

**Table 2:** Mutations in constitutive *RGT2* mutants

*RGT2* mutant designation	Causative constitutive mutation^2^	Location in Rgt2	Other (likely irrelevant) mutations^2^
87	F113L	TM1	
93	F113L	TM1	
58	F113L	TM1	H517R
71	F113L	TM1	T130A, L161S
**82**	D116V	TM1	F562L
68	D116V	TM1	D619G
57	D116G	TM1	I398V, F527L, S754G
100	D116N	TM1	A226T, Y330C
111	D116G, L552S	TM1, ICH5	I111M, V324I, K570R
83	Q222P	TM4	
112	Q222R	TM4	
*RGT2-1*^1^	R231K	TM5	
98	R231G	TM5	D698G
50	R231G	TM5	M58R
103	G232S	TM5	V537A
**59**	G232S	TM5	F465Y, S733L
86	S236P	TM5	F465Y, G471S
**81**	S236P	TM5	K488R
**49^3^**	S253P, S443P	TM5, TM9	
47	D415N	TM8	
60	W474R	TM10	
95	V475M	TM10	
**52**	L552S	ICH5	N723I
85	L552S	ICH5	V404G
108^4^	V473G?		V473G, D611G, I626V
92^4^	?		G103S, F111L

1 The *RGT2*-1 mutation was isolated previously (Ozcan *et al.* 1996).

2 The causative mutations were identified as described in Materials and Methods.

3 Both mutations seem to contribute to constitutive signaling, because each causes a low level of constitutive β-galactosidase activity.

4 We did not attempt to dissect the mutation in these mutants because they are too closely linked.

### Effect of constitutive mutations on glucose transport activity of Hxt1

**Figure 3:**
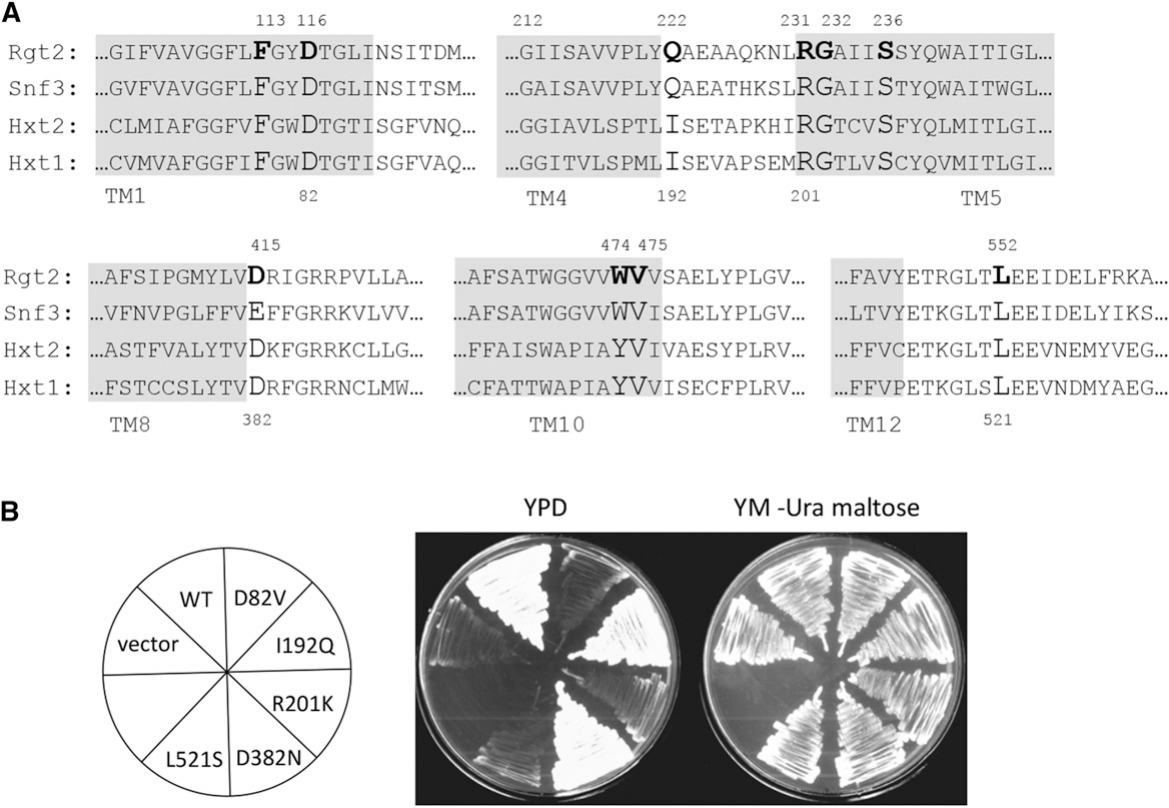
Some of the constitutive mutations alter residues whose orthologs in Hxt1 are required for glucose transport. A) Alignment of amino acid sequences of glucose sensors (Rgt2 and Snf3) and glucose transporters (Hxt1 and Hxt2) in the regions containing the amino acid substitutions responsible for constitutive signaling by Rgt2. B) Plasmids encoding Hxt1 with amino acid changes orthologous to those altered by the constitutive *RGT2* mutations (expressed from the *ADH1* promoter on a multicopy plasmid; see Table 1) were introduced into a strain lacking its glucose transporters (EBY.VW1000) (Wieczorke *et al.* 1999) and tested for the ability to grow on glucose. The mutants plated are shown in the left panel. EBY.VW1000 has functional maltose transporters allowing its growth on YM-maltose.

We tested the effect of five of the constitutive *RGT2* mutations on glucose transport activity of Hxt1, four of which alter well conserved residues in hexose transporters (Figure 3A and 3B, and supplementary Fig Y1). The orthologous amino acids in Hxt1 were changed to the residue resulting from an *RGT2* mutation, and the resulting *HXT1*-containing plasmid was introduced into a yeast strain lacking hexose transporters (Wieczorke *et al.* 1999), which requires a functional glucose transporter to grow on medium with glucose as the sole carbon source (Figure 3B). Three of the 5 mutations cause a glucose transport defect in Hxt1 (Figure 3B, Table 4): Hxt1 mutations D82V, R201K, and L521S, orthologous to Rgt2 mutations D116V, R231K, and L552S, respectively. This suggests that these Hxt1 mutants are unable to carry out a complete transport cycle that leads to an inward-facing conformation and release of glucose into the cell. The Hxt1 D382N substitution, orthologous to Rgt2 D415N, allows glucose transport, suggesting that it is able to carry out a transport cycle and release glucose from the inward-facing structure.

We believe these three mutations directly affect the glucose transport function of Hxt1 for three reasons. First, Hxt1-D82V with GFP fused to its C-terminus is localized to the plasma membrane (Supplementary Fig. Y3), similar to that observed with the wild type. This suggests that degradation or mislocalization of Hxt1 are not the cause of the transport defect. Second, orthologous substitutions of the Hxt1-D82 position in XylE (D27A,E,H,N,S) abolish transport of xylose in a cell-based assay (Wisedchaisri *et al.* 2014), most likely by interfering with a salt bridge (see Figure 5B) necessary for maintaining an outward-facing conformation of the transporter. Third, the R160A substitution in XylE, orthologous to Hxt1-R201, eliminates transport of xylose in a cell-based transport assay (Sun *et al.*, 2012).

We identified three constitutively signaling mutations affecting Gln-222, which is conserved in Rgt2 and Snf3 (Table 4, Figure 3A). The orthologous residue in the yeast hexose transporters is either Ile or Leu, in 12 and 5 of the hexose transporters, respectively. Although the Q to I substitution causes Rgt2 to constitutively signal (Figure 2, Table 4), the converse substitution (I192Q) in Hxt1 does not affect its ability to transport glucose (Figure 3B).

### Predicted structural changes caused by constitutive signaling *RGT2* mutations

To understand the effects of the constitutively-signaling mutations on the structure of Rgt2 we performed a Phyre2 search (Kelley *et al.* 2015), which suggested that Rgt2 has a 3D structure similar to those of several monosaccharide transporters: the rat and bovine GLUT5 facilitated fructose transporter (Nomura *et al.* 2015), the human GLUT3 facilitated glucose transporter (Deng *et al.* 2015), the human GLUT1 facilitated glucose transporter (Deng *et al.* 2014; Kapoor *et al.* 2016), the *E.coli Xyl*E proton:xylose symporter (Sun *et al.* 2012; Quistgaard *et al.* 2013; Wisedchaisri *et al.* 2014), and the *Staphylococcus epidermis* GlcP_Se_ proton:glucose symporter (Iancu *et al.* 2013). While the sequence homology among these proteins is only 20–30% (sequence alignment in Supplementary Fig. Y1), their overall structures are remarkably similar (structural alignment in Supplementary Fig. Y2), suggesting that a reliable model of the Rgt2 structure can be obtained by comparison to them.

**Figure 4:**
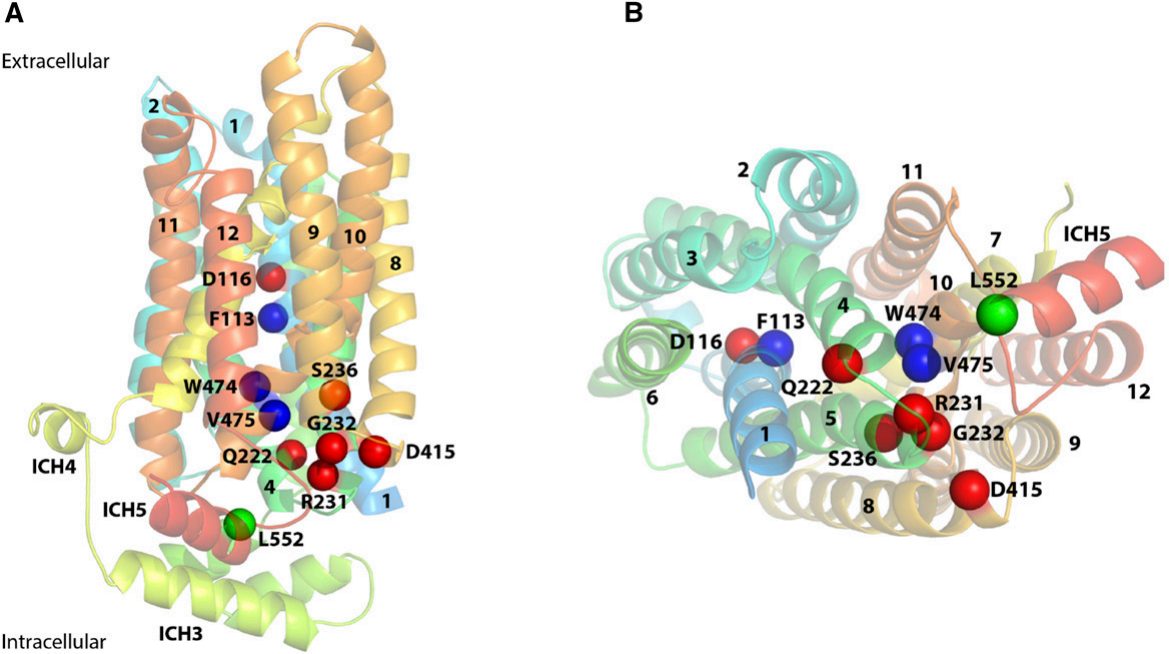
3D model of Rgt2 showing the location of amino acids substitutions in the constitutively signaling mutants. Overview illustrations showing positions of *RGT2* constitutive mutations in a model of Rgt2 generated at the Phyre2 web portal using the rat GLUT5 structure in an outward open conformation (Nomura *et al.* 2015) as template. A) Side view showing the position of mutants located at salt bridge networks (red spheres), in the binding pocket (blue spheres) and at ICH5 (green sphere). B) Cytoplasmic-view showing positions of mutants using the same color coding. Intracellular helices ICH1 through ICH4 are not shown in the cytoplasmic view for clarity.

We used the outward-facing structure of rat GLUT5 to generate a homology model using Phyre2 (Kelley *et al.* 2015) (Figure 4). Positioning Rgt2 residues affected in the 10 constitutive mutants in the predicted structure reveals that nine of the mutations are located in transmembrane spanning helixes; the remaining mutation (L552S) is located at the beginning of intracellular helix 5 (ICH5) (Figure 4). The mutations cluster in the cytoplasmic face of Rgt2, most at the membrane interface.

**Figure 5:**
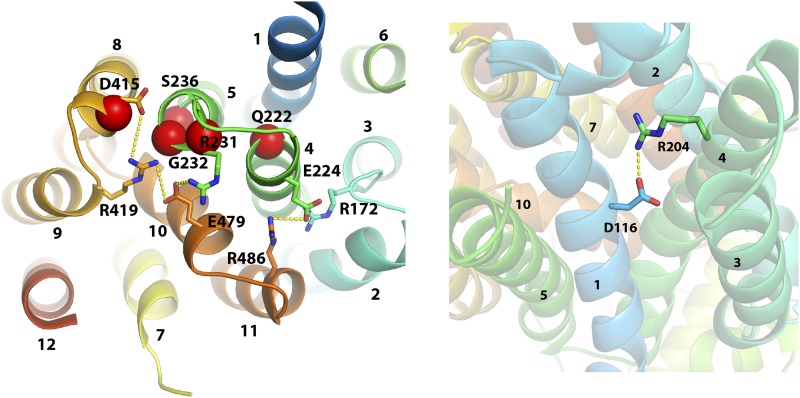
Structural effects of Rgt2 mutantions. A) Mutations interfering with conserved salt bridges at the intracellular gate. Cytoplasmic view of the Rgt2 model showing conserved inter-TM salt bridges that stabilize the outward-facing conformation in the absence of substrate (Nomura *et al.* 2015). Side chains engaged in salt bridges are shown as sticks and salt bridges as dotted lines in yellow. Five mutant positions are indicated by red spheres. Salt bridge network 1 between D415 (TM8), R419 (TM9), E479 (TM10), and R231 (TM5) are directly disrupted by the mutant substitutions R231K, R231G and D415N. The G232S and S236P substitutions may also interfere with salt bridge network 1 due to their close proximity to the bridges. Similarly, the substitution of Q222 (TM4) with either proline, arginine or isoleucine may interfere with salt bridge network 2 between R172 (TM3), E224 (TM4) and R486 (TM11). B) Mutation interfering with the salt bridge between D116 and R204 at the extracellular gate. Extracellular view showing a salt bridge (dotted line in yellow) between D116 in TM1 and R204 in TM4. When the salt bridge is eliminated by the D116V substitution, an inward-facing structure is favored (see Discussion). TM6 (residues G261 to F285) are removed for clarity.

Six of the constitutive mutations alter amino acids at or adjacent to conserved residues that engage in salt bridge interactions in the outward-facing conformations of GLUT5 (Nomura *et al.* 2015), XylE (Quistgaard *et al.* 2013) and GlcP_Se_ (Iancu *et al.* 2013) (see amino acid sequence alignments in Supplementary Fig. Y1). The conserved salt bridge networks found in GLUT5 are shown in the Rgt2 model in Figure 5A; *i.e.*, network 1 with salt bridges between residues D415 (TM8)-R419(TM9)-E479(TM10)-R231(TM5) and network 2 with salt bridges between residues E224(TM4)-R486(TM11)-R172(TM3). As seen in Figure 5A, Rgt2 R231 (TM5) and D415 (TM8) are predicted to be part of salt bridge network 1, and G232 and S236 (TM5) are in its vicinity (Figure 5A). Similarly, Q222 (TM4) is located adjacent to E224, which is participating in salt bridge network 2 (Figure 5A). D116 (TM1) is part of another salt bridge in XylE and GlcP_Se_ that is thought to stabilize their outward-open conformation (Figure 5B) (Henderson and Baldwin 2013; Iancu *et al.* 2013; Wisedchaisri *et al.* 2014; Ke *et al.* 2017).

**Figure 6:**
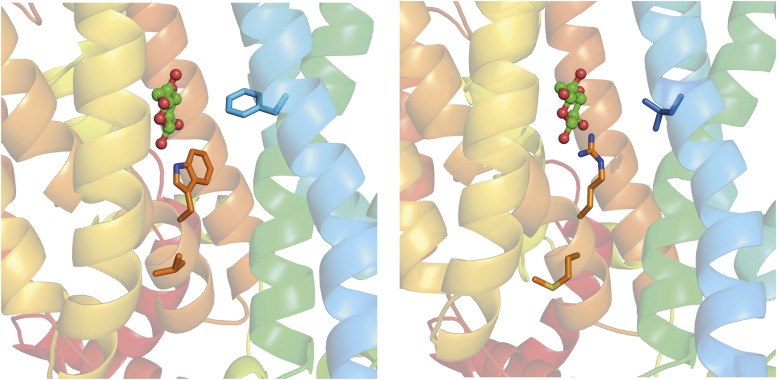
Rgt2 mutations in the sugar binding pocket. Side-view showing a close-up of the binding pocket with a bound glucose molecule. A) The three Rgt2 mutant positions, F113 in TM1, W474 and V475 in TM10 are indicated as sticks. Residues from L230 to Q256 are removed for clarity. The position of the glucose molecule was aligned into the Rgt2 model using XylE with glucose bound in an outward-facing, partly occluded conformation (PDB 4GBZ). B) The substitutions (F113L, W474R and V475M) in the three Rgt2 binding pocket mutants are shown as sticks. The amino acid substitutions may dramatically change side chain interactions in the substrate binding site, thereby promoting a conformational shift toward an inward-open or occluded signaling conformation.

Rgt2 residues F113 (TM1) and W474 (TM10) are located in the substrate binding pocket, and V475 is immediately adjacent to it (Figure 4A, Figure 6). Several lines of evidence support the notion that the constitutive mutations affecting these residues affect substrate binding: 1) The residues are highly conserved in sugar transporters (Supplementary Fig. Y1), and in XylE and GLUT3 the equivalent residues are near the bound glucose molecule and participate in its binding (Sun *et al.* 2012; Deng *et al.* 2015; Yan 2017); 2) substitution of the amino acid in GLUT5 orthologous to Rgt2 F113 results in weak fructose binding (Nomura *et al.* 2015); 3) the amino acid in GLUT1 orthologous to Rgt2 W474 is a key binding determinant for inhibitors (Kapoor *et al.* 2016); 4) changing the W392 residue in XylE orthologous to Rgt2 W474 to Ala almost abolishes transport of xylose (Sun *et al.* 2012); 5) the residues of GLUT1, GLUT3 and GLUT5 that are orthologous to W474 and V475 of Rgt2 are part of the mobile C-terminal transmembrane helix 10b (TM10b), which undergoes local conformational changes during the transport cycle (Deng *et al.* 2014; Deng *et al.* 2015; Nomura *et al.* 2015). Importantly, TM10b and TM4 make cavity-closing contacts, and thereby form an intracellular gate that is believed to open in a substrate-induced manner by moving TM10b away from TM4 and allowing release of the substrate into the cell (Nomura *et al.* 2015; Yan 2015).

**Figure 7:**
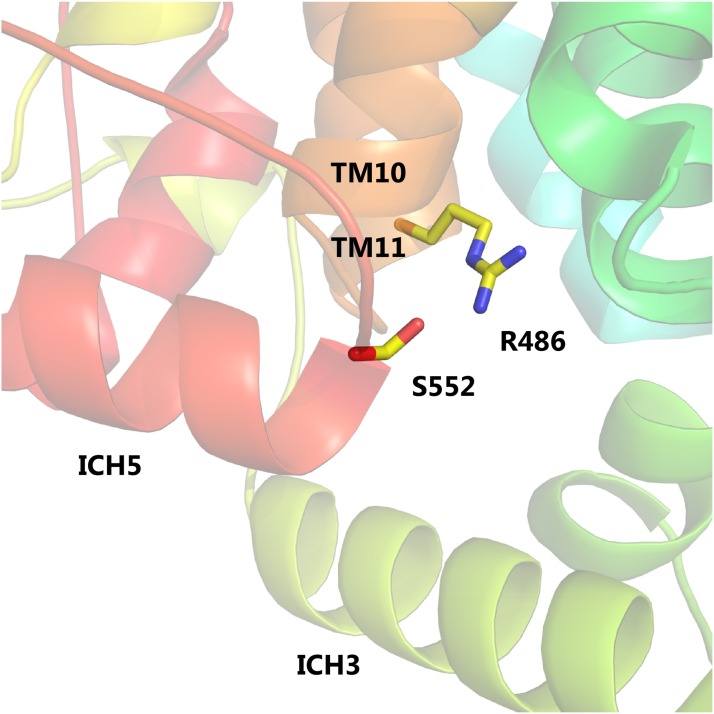
Rgt2 mutation in intracellular helix 5 (ICH5). The Rgt2 mutant that results in substitution of leucine 552 with a serine is shown as sticks. The proximity of S552 in ICH5 to R486 in TM11 is shown. R486 participates in salt bridge network 2 (R172 (TM3), E224 (TM4) and R486 (TM11) (see Fig. 5), and the L552S substitution may change side chain interactions locally to interfere with the salt bridge network and promote a shift toward an inward-open or occluded signaling conformation.

Rgt2 L552, immediately following TM12, is located at the N-terminus of ICH5, part of the ICH domain of sugar porters (Figures 2, 3 and 4A). The ICH domain is proposed to function as a scaffold that helps stabilize the outward-facing conformation (Sun *et al.* 2012; Deng *et al.* 2014; Nomura *et al.* 2015). The Rgt2 L552S substitution may prevent interactions within the ICH domain and/or interactions between the ICH domain and the cytoplasmic ends of the N-bundle and the C- bundle, promoting an inward-facing conformation (Figure 7).

Thus, most of the constitutive mutations interfere with salt bridges that stabilize the outward-facing conformation, and are therefore expected to favor the inward-facing or occluded conformation. Consistent with this notion is the observation that GLUT1 carrying the equivalent of Rgt2 D415N (E329Q in GLUT1) was captured in an inward-facing structure (Deng *et al.* 2014). The three mutations of the glucose-binding pocket may have a similar effect, with the substitutions of W474 and V475 promoting the opening of the intracellular gate from the binding pocket, similar to the release cavity observed in the inward-facing conformation of XylE and GLUT1 (Deng *et al.* 2014; Wisedchaisri *et al.* 2014). These results suggest that the glucose signaling form of Rgt2 is the inward-facing and/or occluded conformation(s).

## Discussion

The Snf3 and Rgt2 glucose sensors are clearly derived from glucose transporters, but differ from them in two significant ways. Most obviously, their long C-terminal tails (337 and 213 amino acids in Snf3 and Rgt2, respectively), which are predicted to reside in the cytoplasm, set them apart from glucose transporters, nearly all of which have tails of less than 50 amino acids. The predominant region of similarity of the Snf3 and Rgt2 tails is the 21 amino acid conserved “box 1” (two copies of which are present in Snf3), which is required for glucose signaling and for interaction with Mth1 and Std1 (our unpublished observations).

The glucose sensor tails are not required for generating the glucose signal, but serve to enhance signaling. This idea is based on our observation that a sensor lacking its tail can generate a glucose signal provided it is overexpressed ((Moriya and Johnston 2004), and Table 3). The fact that none of the *RGT2* mutations we identified that cause constitutive signaling affects the Rgt2 tail is consistent with this idea. (L552 is not considered to be part of the tail because it is immediately downstream of TM12, in a region homologous to sugar transporters (Figures 1-3).) We believe the tails of the glucose sensors simply serve to bring Mth1 and Std1 to the sensors, making them available for phosphorylation, possibly catalyzed by Yck1/2. The critical signaling event, we believe, is the activation of Mth1 and Std1 for phosphorylation when glucose binds to the sensors.

**Table 3:** Glucose-induction of *HXT1* expression by Rgt2

Plasmid	Encoded protein	*HXT1-lacZ* induction (% of wild-type cells)
pBM2974	None (vector)	1
pBM4528	Rgt2 full-length	100
pBM4590	Rgt2 ∆tail a	57
pBM4531	Rgt2 ∆tail b	61
pBM4436	Hxt1	3

The indicated proteins were tested for their ability to mediate induction of *HXT1-lacZ* expression by 4% glucose. The strain YM6212 (Δ*snf3* Δ*rgt2*) carries *HXT1*::*lacZ* (pBM2636) and one of the plasmids indicated. These truncated proteins can mediate glucose signaling because they are expressed at high levels (from the *ADH1* promoter on a multicopy plasmid), as shown previously (Ozcan *et al.* 1998; Moriya and Johnston 2004).

**Figure 8:**
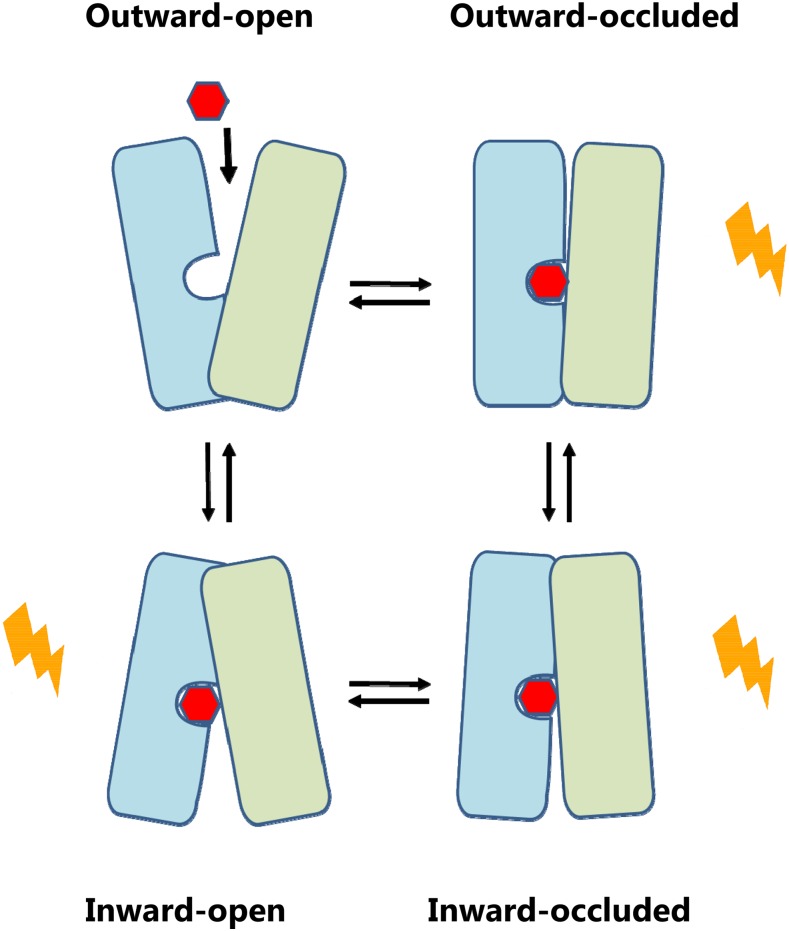
Potential Rgt2 signaling conformation(s). A schematic model for the rocker-switch alternating-access mechanism of sugar transporters. Potential Rgt2 signaling conformations are indicated by a lightning icon. The N-helix bundle (TM1-6) is colored green; the C-helix bundle (TM7-12) is colored blue. The substrate binding site is illustrated as a cavity in the C-bundle, since this domain provides the majority of residues that coordinate glucose binding through hydrogen bonds, as revealed in, for example, GLUT3 (Deng *et al.* 2015). A red hexagon symbolizes the sugar. The model is based on published structures: Outward-facing structures: Rat GLUT5 fructose transporter without substrates, in complex with single chain Fv fragment (Protein Data Bank ID 4YBQ); human GLUT3 glucose transporter with bound maltose (4ZWC). Outward-occluded structures: *E.coli* XylE xylose transporter with bound xylose, glucose or 6-Br-glucose (4GBY, 4GBZ and 4GCO, respectively); human GLUT3 glucose transporter with bound glucose or maltose (4ZW9 and 4ZWB, respectively). Inward-occluded structure: *E.coli* XylE without substrate (4JA3). Inward-facing structures: *E.coli* XylE without substrate (4JA4 and 4QIQ); *S. epidermis* GlcP glucose transporter without substrate (4LDS); human GLUT1-E329Q glucose transporter with bound βNG (4PYP); human GLUT1 with bound inhibitors (5EQI, 5EQG and 5EQH); bovine GLUT5 fructose transporter without substrate (4YB9).

The glucose sensors and glucose transporters differ in the way they handle glucose: the glucose sensors cannot transport glucose (Figure 1B), but they generate the signal that leads to induction of *HXT* gene expression. We believe this functional difference is due to differences in the sequences of the transmembrane domains of the glucose sensors, since the sensor tails do not seem to affect glucose transport (Figure 1B) and are not required for signaling (Moriya and Johnston 2004). We think of the sensors as former glucose transporters that accumulated sequence changes during evolution that prevent the conversion of their glucose-binding site from the outward-facing conformation to the inward-facing configuration that delivers glucose into the cell (Figure 8) (Yan 2015; Drew and Boudker 2016; Yan 2017). Instead, the conformational change induced by binding of glucose to the sensors has been diverted to activate phosphorylation of Mth1 and Std1.

Our Phyre2 analysis of Rgt2 suggests it is structurally similar to known sugar porters XylE, GlcP, GLUT1, GLUT3 and GLUT5, so we expect that Rgt2 can assume conformations related to the conformations of genuine sugar porters along their transport cycle (Figure 8). It seems likely that glucose binds to the outward-facing conformation of Rgt2 and causes it to attain a signaling conformation without the release of glucose into the cell.

Seven of the constitutively-signaling *RGT2* mutations we identified likely interfere with maintaining the outward-facing Rgt2 structure in the absence of glucose. Five of them are clustered at the intracellular face of Rgt2 (Figure 4); they probably affect the network of interbundle salt-bridges that are highly conserved in monosaccharide transporters, from bacteria to mammals (Quistgaard *et al.* 2013; Deng *et al.* 2015; Nomura *et al.* 2015; Drew and Boudker 2016). These salt bridges stabilize the outward-facing conformation by bringing together the cytoplasmic ends of the N- and C-bundles in the absence of substrate. Substitutions in Rgt2 of R231 in TM5 and D415 in TM8 directly disrupt the salt-bridges (Figure 5A). Because an E329Q substitution in GLUT1 locks it in an inward-open conformation (Deng *et al.* 2014), we suggest that the orthologous D415N Rgt2 mutation also causes Rgt2 to adopt the inward-facing conformation. We propose that the Rgt2 G232 and S236 mutations, which are located in TM5 in the vicinity of salt bridges (Figure 5A), disrupt salt bridges and promote an inward-facing conformation.

Residue Q222 in TM4 is changed to proline, arginine or isoleucine in three independent constitutive *RGT2* mutants. This residue, at the intracellular tip of TM4 (Figure 4A), may influence formation of neighboring salt bridges and/or the position of the TM4 and TM10 gating helices, which are in close contact in the outward-facing conformation (Yan 2015; Drew and Boudker 2016). Thus, these mutations may cause TM4 and TM10 to move apart, thereby facilitating the inward-facing conformation.

Residue D116, located in TM1 at the extracellular face of Rgt2, probably forms a salt bridge to R204 in TM4 (Figure 5B), similar to salt bridges between orthologous residues D27 and R133 in XylE (Sun *et al.* 2012; Henderson and Baldwin 2013; Quistgaard *et al.* 2013; Wisedchaisri *et al.* 2014), and between D22 and R102 in GlcP_Se_ (Iancu *et al.* 2013). The Rgt2 D116 residue is conserved in Snf3, as well as in the 17 related sugar transporters of *S. cerevisiae*, and the Rgt2 R204 residue is invariant in orthologous positions in all sugar porters. Disruption of the salt bridge between these aspartic acid and arginine residues in XylE and GlcPSe abolishes active transport (Iancu *et al.* 2013; Madej *et al.* 2014; Wisedchaisri *et al.* 2014), emphasizing the importance of this salt bridge in sugar transport. Similarly, we find that the D82V substitution in Hxt1, orthologous to Rgt2 D116, causes a glucose transport defect (Figure 3B). The importance of the D116-R204 salt bridge for maintaining the outward-open conformation was recently revealed using a PEGylation assay and bioinformatic analysis (Ke *et al.* 2017).

XylE and GlcPSe are symporters that transport sugars along with a proton. Protonation of D27 in XylE or D22 in GlcPSe is believed to break their interaction with an arginine residue and trigger the outward-to-inward transition that releases the sugar into the cytoplasm (Iancu *et al.* 2013; Wisedchaisri *et al.* 2014; Yan 2015). Yeast sugar porters catalyze the movement of sugars down a concentration gradient by facilitated diffusion, without proton symport (Bisson *et al.* 2016). Our results suggest that the Rgt2 D116V substitution in TM1 eliminates its interaction with R204 in TM4, leading to a signaling conformation, perhaps by changing the position of TM4 such that the intracellular cavity-closing contacts between the gating helices TM4 and TM10b are disrupted.

Additional stabilization of the outward-facing conformation of sugar transporters is also provided by four intracellular helices (ICH1-4) in the cytoplasmic loop between TM6 and TM7, and ICH5 that follows TM12 in the cytoplasm (Sun *et al.* 2012; Deng *et al.* 2014; Nomura *et al.* 2015). Because the Rgt2 L522S mutation is located at the N-terminal end of ICH5 (Figure 4A), we speculate that this mutation may affect the ICH stabilization such that the N-helix and C-helix bundles move apart and opens the intracellular gate, resulting in an inward-facing structure. In GLUT5 a salt bridge between E252 in ICH3 and R407 in TM11 helps stabilize the outward-facing structure (Nomura *et al.* 2015). GLUT5 R407 is orthologous to Rgt2 R486, which is part of an inter-bundle salt-bridge network maintaining the outward-facing form (Figure 5A). Since L552 and R486 are in close proximity in our Rgt2 model (Figure 7), it is tempting to speculate that the L552S substitution interferes with this salt bridging. The homologous L552S substitution in Hxt1 abolishes transport, emphasizing that this substitution significantly influences both transport (Figure 3B) and signaling (Table 4).

**Table 4:** Phenotypes of *RGT2* constitutive mutations

Rgt2 aa	Rgt2 location	aa change	# isolated	*HXT1* induction^1^	Orthologous position in Hxt1	glucose transport by mutant Hxt1
F113	TM1	L	4	7.3		
D116	TM1	V	2	12.1	D82V	—
D116	TM1	G	2			
D116	TM1	N	1			
Q222	TM4-5	P	1	10.1		
Q222	TM4-5	R	1			
Q222	TM4-5	I	*	11.3	I192Q	+
R231	TM4-5	G	2			
R231	TM4-5	K	*RGT2-1*	12.8	R201K	—
G232	TM5	S	2	9.7		
S236	TM5	P	2	9.1		
D415	TM8-9	N	1	12.5	D382N	+
W474	TM10	R	1	10.1	Y449	—
V475	TM10	M	1	11.7		
L552	ICH5	S	3	12.1	L521S	—

1 *HXT1* expression in cells grown on 5% glycerol + 0.5% galactose (relative to expression in cells with wild-type *RGT2* = 1).

A detailed understanding of the substrate binding site of sugar porters has been revealed from the 1.5 Å structure of GLUT3 in complex with glucose (Deng *et al.* 2015). The structure shows that glucose is coordinated in the substrate binding site by seven polar residues mainly from the C-bundle and three hydrophobic residues from each of the N- and C-bundles (Deng *et al.* 2015; Yan 2017). As explained in the Results section, the Rgt2 mutants F113L, W474R and V475M lie in this well-defined substrate binding site (Figure 4A and 4B, Figure 6). The binding site is expected to be involved in shifting the conformation from the outside to the inside orientation upon substrate binding, as discussed in recent review articles (Yan 2015; Drew and Boudker 2016; Yan 2017). The Rgt2 W474R and V475M substitutions alter residues in the substrate binding site in helix TM10b (Figure 4A and 4B, Figure 6) which makes intracellular cavity-closing contacts to TM4 and contributes to formation of the intracellular gate. The gate is disrupted during the glucose release process from the inward-open conformation, which is accompanied with an outward swing of TM10b. Interestingly, it is suggested that the residue orthologous to Rgt2 W474 in GLUT3 (W386) looses contact to glucose during these local structural shifts (Deng *et al.* 2015), and mutation of the orthologous Trp in XylE (W392A) abolishes transport (Sun *et al.* 2012). Thus, we reason that the W474R and V475M substitutions induce local movements of gating helix TM10b leading to an opening of the intracellular gate, similar to the substrate-induced movement of the helices, resulting in an inward-facing conformation.

The F113 residue in Rgt2 is evolutionarily conserved in yeast and human hexose transporters. The orthologous residues in GLUT3 and GLUT5 (F24 and Y31, respectively), which are located in the middle of TM1, are considered central cavity residues that participate in substrate binding. The structure of GLUT3 reveals that F24 interacts with the carbon backbone of the bound D-glucose sugar ring (Deng *et al.* 2015), and an Y31A substitution in GLUT5 results in weak fructose binding (Nomura *et al.* 2015). Similarly, the orthologous F24 in XylE is located in the vicinity of the substrate, and an F24A substitution causes significant reduction of transport activity (Sun *et al.* 2012). In our Rgt2 model (Figure 4A, Figure 5C), F113 is located close to the bound glucose, and we infer that the F113L substitution may “mimic” glucose binding and facilitates a transition toward an inward-facing conformation, similar to the rearrangements that occur during the glucose transport process.

Hxt1 with substitutions orthologous to the constitutively-signaling D116V, R231K, or L552S mutations in Rgt2 is defective in glucose transport (Figure 3B). Hxt1 with these mutations must be unable to carry out a complete transport cycle that leads to the release of glucose into the cell (Figure 8). Because our analysis suggests that the constitutive mutations cause Rgt2 to adopt an inward-facing conformation, we imagine the D116V, R231K, or L552S *HXT1* mutations lock Hxt1 in an inward-facing conformation that does not allow binding of extracellular glucose, or in a substrate-bound occluded conformation that is unable to proceed to an inward-facing conformation. Interestingly, GLUT1 mutations affecting residues orthologous to Rgt2 R231 (GLUT1 R153) and D415 (GLUT1 E329) are also found in people with GLUT1-deficiency disease in which glucose transport into the brain is impaired (Leen *et al.* 2010).

The Rgt2 D415N substitution is something of an enigma. The orthologous D382N substitution in Hxt1 does not affect its ability to transport glucose (Figure 3B). Several observations support the importance of this residue in other sugar transporters: 1) the orthologous E329Q substitution in human GLUT4 was suggested to arrest this transporter in an inward-facing conformation (Schürmann *et al.* 1997); 2) human GLUT1 with an E329Q substitution crystallized in an inward-open conformation in a complex with β-NG (Deng *et al.* 2014), and has reduced ability to transport glucose into the brain (Leen *et al.* 2010); 3) a D337L substitution of *E. coli* XylE favors an inward-facing conformation (Ke *et al.* 2017). Thus the overall picture is that mutations affecting the residue orthologous to Rgt2 D415 favor the inward-facing conformation and compromise glucose transport function. The orthologous D382N substitution in *HXT1* may favor the inward conformation of Hxt1 without preventing its oscillation back to the outward-facing conformation.

Our analysis of constitutively-signaling *RGT2* mutations suggests that the mutations destabilize the outward-facing conformation and promotes an inward-facing conformation of Rgt2, which is its signaling conformation. This would seem to conflict with the observation that high levels of intracellular glucose inhibits signaling by Snf3, because that was assumed to shift the equilibrium of the sensor to the inward-facing form (Karhumaa *et al.* 2010). But we would argue that binding of glucose to the intracellular face of the sensors favors their outward-facing (non-signaling) form, and that glucose bound to the extracellular face induces the inward-facing (signaling) form of the sensors. Or perhaps it is the occluded (intermediate) forms of the sensors that are responsible for generating the glucose signal, since glucose is not transported by Rgt2 and Snf3. In any case, resolution of this must await solution of the Rgt2 structure. The constitutive *RGT2* mutations that lock the sensor in its signaling form(s) promise to facilitate its crystallization necessary to determine its structure.

All of our constitutive *RGT2* mutations cause only partial signaling by the glucose sensor: they cause an average 10.8-fold induction of *HXT1* expression (range 7.3 – 12.8-fold; Table 4), while glucose induces *HXT1* expression approximately 100-fold in wild-type cells (Moriya and Johnston 2004). Indeed, *HXT1* expression in the *RGT2-1* mutant is induced approximately 10-fold by glucose (Table 2 in (Ozcan *et al.* 1996). Thus, the constitutive Rgt2 sensors seem to be about 10% efficient at generating a signal. (But note that some of the additional 90% induction of *HXT1* expression by glucose is due to two other pathways that regulate *HXT1* expression—the high osmolarity sensing Hog1 pathway (Tomás-Cobos *et al.* 2004), and a pathway that involves the hexokinase Hxk2 (Belinchón and Gancedo 2007)—so the constitutively-signaling sensors are likely to be somewhat more than 10% efficient at generating a signal.) This is perhaps not surprising because of the relatively large number of interactions that stabilize the outward-facing (non-signaling) form of the sensor. Apparently, any single amino acid change only partially destabilizes the outward-facing form.

It has been proposed that human glucose transporters may play roles as sugar sensors, also referred to as transceptors (Thevelein and Voordeckers 2009). The intestinal glucose transporter SGLT1 functions as a sensor that mediates release of gastrointestinal peptide hormones into the circulation (Diez-Sampedro *et al.* 2003; Daniel and Zietek 2015). Glucose detection by the glucose transporter GLUT2 contributes to the control of food intake by the hypothalamus (Stolarczyk *et al.* 2010), and GLUT2 may be involved in stimulation of pancreatic β-cell differentiation and insulin secretion (Michau *et al.* 2013). Thus, studies of Rgt2 may inform glucose sensing in mammals.
